# Alternatives to mineral oil adjuvants in vaccines against *Aeromonas salmonicida* subsp. *salmonicida* in rainbow trout offer reductions in adverse effects

**DOI:** 10.1038/s41598-017-06324-7

**Published:** 2017-07-19

**Authors:** Kasper Rømer Villumsen, Erling Olaf Koppang, Dennis Christensen, Anders Miki Bojesen

**Affiliations:** 1 Department of Veterinary and Animal Sciences, University of Copenhagen, Copenhagen, Denmark; 2 Faculty of Veterinary Medicine, Norwegian University of Life Sciences, Oslo, Norway; 3 Adjuvant Research, Statens Serum Institut, København S, Denmark

## Abstract

In an effort to reduce the frequency and severity of adverse reactions seen from the use of mineral oil adjuvants in salmonid fish, the effects of two alternative adjuvants were assessed, focusing on the induction of adverse effects as well as protection. Using rainbow trout (*Oncorhynchus mykiss*) as recipients, injection vaccines based on formalin-inactivated *Aeromonas salmonicida* subspecies *salmonicida* were formulated with CpG oligodeoxynucleotides, the liposomal cationic adjuvant formulation 01 (CAF01) or with Freund’s incomplete adjuvant and administered intraperitoneally. Control groups of unvaccinated, Tris-buffered saline-injected or bacterin-injected individuals were included, and each group included in the study held a total number of 240 individuals. Subsequently, individuals from each group were examined for differences in Fulton’s condition factor, macro- and microscopic pathological changes, as well as protection against experimental infection with *A. salmonicida*. While adverse effects were not eliminated, reductions in microscopic and macroscopic adverse effects, in particular, were seen for both the nucleotide- and liposome-based vaccine formulations. Furthermore, the induced protection appears similar to that of the benchmark formulation, thus introducing viable, potential alternative types of adjuvants for use in future fish vaccines.

## Introduction

Intensive, modern aquaculture production depends on successful prophylactic control of and treatment against a variety of waterborne viral, parasitic, as well as bacterial pathogens. High stocking densities and an increasingly intense production have led to a continuous demand for effective vaccine strategies in order to ensure protection, minimizing loss as well as the use of antibiotics during the production cycle.

Furunculosis caused by the Gram-negative bacterium *Aeromonas salmonicida* subsp. *salmonicida* is a threat to the production of salmonid fish species, including Atlantic salmon (*Salmo salar*) and rainbow trout (*Oncorhynchus mykiss*). Current prophylactic strategies rely primarily on intraperitoneal injection of vaccines formulated with mineral oil adjuvants. This strategy has successfully induced long-term protection^1–5^, however, a variety of adverse effects, including adhesions and pigmentation of the peritoneal viscera, growth reduction^2, 3, 5, 6^, deformed vertebrae^7^, autoimmunity and hypergammaglobulinemia^8, 9^ have been observed. Such reactions affect animal welfare as well as the quality of fish products. Therefore, a continued effort to improve the current vaccine formulations is warranted, as an ideal vaccine should provide optimal protection, while simultaneously inducing minimal adverse effects.

In order to address these criteria from an adjuvant perspective, three different experimental vaccine formulations were prepared using identical bacterial antigens: a benchmark formulation with Freund’s incomplete adjuvant (FIA) representing the standard mineral oil adjuvanted formulation, and two alternative, experimental formulations based on CpG oligodeoxynucleotides (CpG ODNs) and the liposomal adjuvant Cationic Adjuvant Formulation 01 (CAF01), respectively.

Unmethylated CpG ODNs have been shown to have immunostimulatory effects in mammals as well as teleosts, mediated through Toll-like Receptor (TLR) 9 signaling^10, 11^ and the methylation status of bacterial DNA as well as the integrity of the CpG motifs have been shown to influence the induction of B-cell proliferation and IgM secretion^12^. CpG ODN’s have been tested in human clinical trials as an adjuvant in several vaccine formulations (see ref. 13). The CAF01 adjuvant system consists of dimethyldioctadecylammonium bromide liposomes supplemented with the synthetic mycobacterial cord factor α,α-trehalose 6,6-dibehenate^14, 15^. Designed for use in humans, CAF01 has previously been shown to promote a mixed Th1/Th17 profile in murine models when used with single-peptide antigens from a number of different pathogens^16, 17^. Furthermore, induction of protection against the human pathogens *Mycobacterium tuberculosis* and *Chlamydia trachomatis* has been demonstrated using this adjuvant^16^. Vaccine formulations utilizing CAF01 as an adjuvant for use in humans have been shown to exhibit a satisfactory safety profile^18^, and have advanced to clinical trials^19^. Freund’s incomplete adjuvant was included to serve as a benchmark formulation, based in part on its chemical composition of mineral oil and surfactant that resembles that of commercially available vaccines, and also on previous experiences with induction of both protection and adverse effects with this formulation in rainbow trout^4, 5^. The water-in-oil characteristics of FIA emulsions, are thought to contribute to a depot-effect through which antigen is released slowly over time, and also to contribute to increased uptake by phagocytic cells^20^.

In keeping with the previously stated criteria, the *in vivo* application of each formulation was examined with regards to condition, macro- and microscopic pathological changes following intraperitoneal administration to rainbow trout, and finally an experimental, homologous infection study was performed to assess the protection offered by each formulation.

## Materials and Methods

### Ethics Statement

The animal study protocols, as well as the methods involving handling or treatment of the animals described in the present study, were approved by the Danish Animal Experiments Inspectorate under the Danish law on animal experiments. The study was performed under license no. 2014-15-0201-00252.

### Fish

Rainbow trout (*Oncorhynchus mykiss*) eggs (all-female, AquaSearch FRESH, Fousing strain, AquaSearch ova ApS, Billund, Denmark) were disinfected using Actomar K30 (according to the manufacturers instructions), hatched in late November 2014 and reared at AquaBaltic (Nexø, Denmark). After hatching, the fish were kept in fiberglass tanks (500 l) at 15 °C in a fully recirculated system (20 l/min/tank) with a light/dark cycle of 5 h/19 h. The fish were fed commercial pelleted feed (BioMar, Denmark) corresponding to 0.5% of the total fish weight/tank.

### Vaccines and Experimental Group Setup

All experimental vaccines were formulated with the same bacterin consisting of formaldehyde-inactivated *Aeromonas salmonicida* subspecies *salmonicida* in combination with one of three adjuvants, as detailed below.

#### Bacterin

A starter culture of *A. salmonicida* ssp. *salmonicida* (040617-1/1A, kindly provided by Inger Dalsgaard, DTU, Denmark) was made from a 20% (v/v) glycerol stock onto 5% blood agar plates (State Serum Institute (SSI), Copenhagen, Denmark), followed by an incubation period (20 °C, 48 h). Colonies on the plate were confirmed as *A. salmonicida* using an agglutination kit (MONO-As, Bionor Laboratories AS, Norway), before transfer into 200 ml filtered (10 µm) and autoclaved Bacto™ heart infusion broth (Becton, Dickinson & Co, USA). The culture was incubated overnight (20 °C, 200 rpm). Growth was monitored by OD_600 nm_ measurement and the culture was finally inactivated by addition of formaldehyde to a final concentration of 10% (v/v) at room temperature. Prior to inactivation, the final CFU/ml was estimated by a modified Miles & Misra method^21^, by counts made from triplicate plating of 10 µl/step in a nine-step, ten-fold dilution series. Furthermore, triplicate 100 µl smears were made on 5% blood agar plates, to assess the homogeneity of the culture. After inactivation, the formaldehyde was removed by centrifugation (4068G, 5 min), followed by three washes in tris-buffered saline (TBS, Sigma-Aldrich, USA). Finally the concentration was adjusted to 2 × 10^9^ CFU/ml in TBS. Triplicate 100 µl smears on 5% blood agar plates were made during, as well as after incubation with formaldehyde to confirm the inactivation.

#### Adjuvants

1. CpG oligodeoxynucleotides (ODN’s). The sequence (Table 1) was based directly on CpG ODN 2143^22, 23^, featuring a 40% GC-content, phosphorothioate bonds throughout the structure and a molecular weight of 8018 g/mol (Eurofins Genomics, Germany). The ODN’s were purified by HPLC and lyophilized prior to shipment.

**Table 1:** Treatment specifications for each experimental group.

Experimental Formulation:	Individuals:	Mix Ratio (v/v):	Content Per 100 µl Dose:	
			*A. salmonicida*	Adjuvant
Naïve controls	240	—	—	—
TBS controls	240	—	—	TBS Only
Bacterin controls	240	—	1 * 10^8^ CFU	TBS Only
Bacterin + CpG ODN	240	1:1	1 * 10^8^ CFU	0.6 nM CpG ODN in TBS
Bacterin + CAF01	240	1:1	1 * 10^8^ CFU	300 µg CAF01 in TBS
Bacterin + FIA	240	1:1	1 * 10^8^ CFU	50 µl FIA

The naïve controls did not receive any injection.
  5′-TTCGTCGTTTTGTCGTTTTGTCGTT-3′
  Prior to use, the ODN’s were resuspended in TBS to a 12 µM working solution. The final dosage is equal to that used by Carrington & Secombes^23^.
2. Cationic adjuvant formulation 01 (CAF01). Liposomal adjuvant consisting of dimethyldioctadecyl-ammonium bromide (DDA) liposomes supplemented with α,α-trehalose 6,6-dibehenate (TDB) (5:1), suspended in TBS (SSI, Denmark)^14, 15, 18^.
3. Freund’s incomplete adjuvant (FIA, 85% paraffin oil and 15% mannide monooleate (v/v)) (Sigma-Aldrich, USA).

#### Control groups

Three control groups of 240 individuals each were included: Bacterin injected, TBS injected and naïve controls. The composition of each formulation included in the study is detailed in Table 1.

### Vaccination

Vaccinations were performed in June 2015. Following the minimum weight requirement of injection vaccines against *A. salmonicida* approved for use in Danish aquaculture, the average weight at the time of vaccination was >15 gr. Feed was withheld for three days prior to vaccination, as recommended by commercial guidelines^24^. Prior to vaccination each experimental formulation was mixed, and the vaccine pistol was rinsed out by multiple ejections of TBS between each experimental formulation. Prior to use, all formulations were thoroughly mixed. The FIA-formulation was emulsified through repeated passages between two syringes through a connector, and prior to use the consistency of the FIA-formulation was tested, and found to be emulsified and not readily miscible with water. Immediately prior to injection, the fish were anesthetized in 100 mg/l tricaine methanesulfonate (MS-222, Sigma-Aldrich, USA). Intraperitoneal injections (100 µl/dose) were made according to commercial vaccine guidelines^24^, using a heat-sterilized FishJector manual vaccine pistol fitted with a 4 mm, 23G syringe (Kaycee Veterinary Products Ltd, UK). Immediately after injection the fish were transferred to fresh water in separate tanks, sorting a total of 240 vaccinated and/or allocated fish to each experimental group.

### Sampling and Gross Pathological Scoring

Prior to vaccination, as well as three and ten weeks post vaccination ten fish/group were randomly netted and euthanized in 200 mg/L MS-222. As a measure of overall fitness of each fish, the weight (in grams) and fork length was recorded (in cm) for calculation of Fulton’s Condition Factor:^25, 26^

1 $$K=Weight/Lengh{t}^{3}$$

At the two latter time points the weight and length measurement was followed by post-mortem gross pathological examination of the intraperitoneal cavity. The cavity was exposed by an incision following the ventral midline, although avoiding the injection site, and carefully examined and scored according to three different gross pathological scoring systems: a Speilberg score, as well as separate scores for pigmentation and adhesions of the viscera, thus recording three separate scores for each individual. Speilberg scoring was performed according to the criteria detailed by Midtlyng *et al*.^3^. The pigmentation and adhesion score both operate on the combined observed extent and severity of either pigmentation or adhesion(s) of the intraperitoneal viscera, as follows: 0): No observations, or 1): Single, 2): Moderate and 3): Extensive pigmentation(s)/adhesion(s). One person performed the scoring of all individuals, however, the examination was not blinded. Next, the anterior kidney, spleen, liver, heart, the site of injection/vaccine deposition (ventral section including intraperitoneal viscera, musculature and scales), as well as any observed pigmentation and/or adhesions following tissues were carefully sampled for histological examination. For non-vaccinated fish, the ventral section corresponding to the injection site was sampled. A streak from the anterior kidney was made using a sterile inoculation loop on a 5% blood agar plate. Immediately after sampling, the tissues were transferred to 10% neutral buffered formalin (4% formaldehyde, CellPath, UK). After 24 h, the formalin solution was replaced with 70% ethanol, and the samples were stored at 6 °C until further processing.

### Assessment of Subclinical Infections

A small piece of each sampled spleen was dissected, placed in RNA*later* (Sigma-Aldrich) and stored at −20 °C. Five spleen samples from each group/tank sampled ten weeks post vaccination were analyzed for presence of *Flavobacterium psychrophilum, Renibacterium salmoninarum, Yersinia ruckeri*, infectious pancreas necrosis virus, piscine reovirus, piscine orthoreovirus 2 (virus Y in trout), pancreas disease virus/salmonid alphavirus and viral hemorrhagic septicemia virus by means of real-time PCR analysis (PatoGen Analyse AS, Ålesund, Norway).

### Histology

Samples for histological examination were stained with hematoxylin and eosin (HE), periodic acid-Schiff-stain (PAS), as well as martius yellow, brilliant crystal scarlet and methyl blue (MSB) (Veterinary Pathology Lab, University of Copenhagen). One paraffin block was prepared from each of the 10 individuals sampled from each group at each sampling time point, with all sampled tissues embedded together. For HE staining, one section per individual was processed for samples 1–10/group/time point. For further special stains, one section was processed for samples 1–2/group/time point for PAS staining, and finally, one section was processed for samples 1–5/group/time point for MSB staining.

### Experimental infection

Fish were transported to the experimental facilities at the University of Copenhagen 84 days post vaccination in September 2015. This corresponded to 1260 degree days. This measure is calculated by multiplying the number of days in a given period by the temperature in degrees Celsius, and provides a way of comparing immunological development in fish kept at varying temperatures. Fish from each experimental group were divided into duplicate 150 l aquaria (≈30/aquarium, see Table 2) equipped with constant aeration and individual, internal biofilters, and allowed to acclimatize for 14 days. The feed was identical to that given at AquaBaltic, and the water temperature, directly controlled by the room temperature, was 20 °C at the onset of the experiment, followed by a decline throughout the infection, as shown in Supplementary Fig. S1. Prior to infection 2 individuals/aquarium were euthanized in 200 mg/l MS-222, and streaks from the anterior kidney were made onto 5% blood agar plates. The plates were incubated at 20 °C for 4 days before examination and were found to confirm the non-infected status of the fish.

**Table 2:** Individuals per aquarium during the experimental infection.

Exp. group	Non-Infected Control	Infected Control	TBS Control	Bacterin Control	CpG-Formulation	CAF01-Formulation	FIA-Formulation
Aquarium 1	29	25	23	27	28	28	28
Aquarium 2	27	30	33	28	28	27	28

For use in the infection experiment, a culture of *A. salmonicida* subsp. *salmonicida* (040617-1/1A) was grown, but not inactivated, as described above, with the exception that 12 l of autoclaved, but unfiltered HIB was used. Again, the sterility of the medium, as well as the homogeneity of the finished culture were confirmed by 100 µl spread culture on 5% blood agar plates. Prior to infection, the culture was washed three times in tap water (4068G, 5 min), and CFU/ml was assessed as described above. Individuals in each aquarium, except for the non-infected group, were transferred to infection tanks and exposed to a waterborne, homologous bath challenge in aerated water (5 l, 1.2 * 10^8^ CFU/ml of *A. salmonicida* subsp. *salmonicida* (040617-1/1A)) for an hour, during which all tanks were carefully monitored. Subsequently, all fish were returned to their respective holding aquaria, and all individuals were examined at least three times/day for the duration of the following 28 day experimental infection. During the expected peak mortality period between three and nine days post infection, the fish were monitored at least four times/day. The following criteria served as humane endpoints: clear signs of disease, such as pronounced solitary behavior, lack of ability to maintain equilibrium and/or occurrence of abscesses. Upon meeting one or more of these criteria, the fish was considered moribund and euthanized in 200 mg/L MS-222. These endpoints were applied throughout the experiment, despite references to “mortality” and “survival” in the present text. These terms merely adhere to standard survival analysis terminology. Once euthanized, the exterior of the individual was carefully cleaned with 70% ethanol, a dorsal incision was made and a streak from the anterior kidney was made on a 5% blood agar plate. These were then incubated at 20 °C, and only individuals from which re-isolation of *A. salmonicida* could be verified using the MONO-As agglutination kit were computed as mortalities. Otherwise the fish were censored in the Kaplan-Meier analysis.

### Statistical analysis

All analyses were performed using GraphPad Prism 6 for Mac (GraphPad Software, Inc). For all comparative analyses, a 95% confidence interval was used and P values < 0.05 resulted in a rejection of the null-hypothesis that there was no statistically significant difference(s) between the groups in question.

Fultons condition factor was calculated as described above. A D’Agostino & Pearson omnibus normality test was applied to test if the data approached a Gaussian distribution. Subsequently, a one-way ANOVA was performed, followed by a Tukey’s multiple comparisons test to identify difference(s) between groups. Only samples obtained at the same time were compared.

The data obtained from the gross pathological scoring was analyzed using a Kruskal-Wallis test followed by a Dunn’s post test for multiple comparisons.

Survival data from the infection study was processed using the Kaplan-Meier method^27^. The criteria for events and censoring are described above. A Mantel-Cox log-rank test was used to compare survival curves. The computed log-rank hazard ratio for each vaccine group relative to the infected control group was derived as well, providing a measure of the relative risk of mortality of each vaccine group when compared to the infected control group. The relative percent survival (RPS) of each group was calculated as follows^28^:

2 $$RPS=(1-(mortality\,in\,treated\,group/mortality\,in\,infected\,control\,group))\times 100 \% $$

## Results

### Fultons Condition Factor

**Figure 1:**
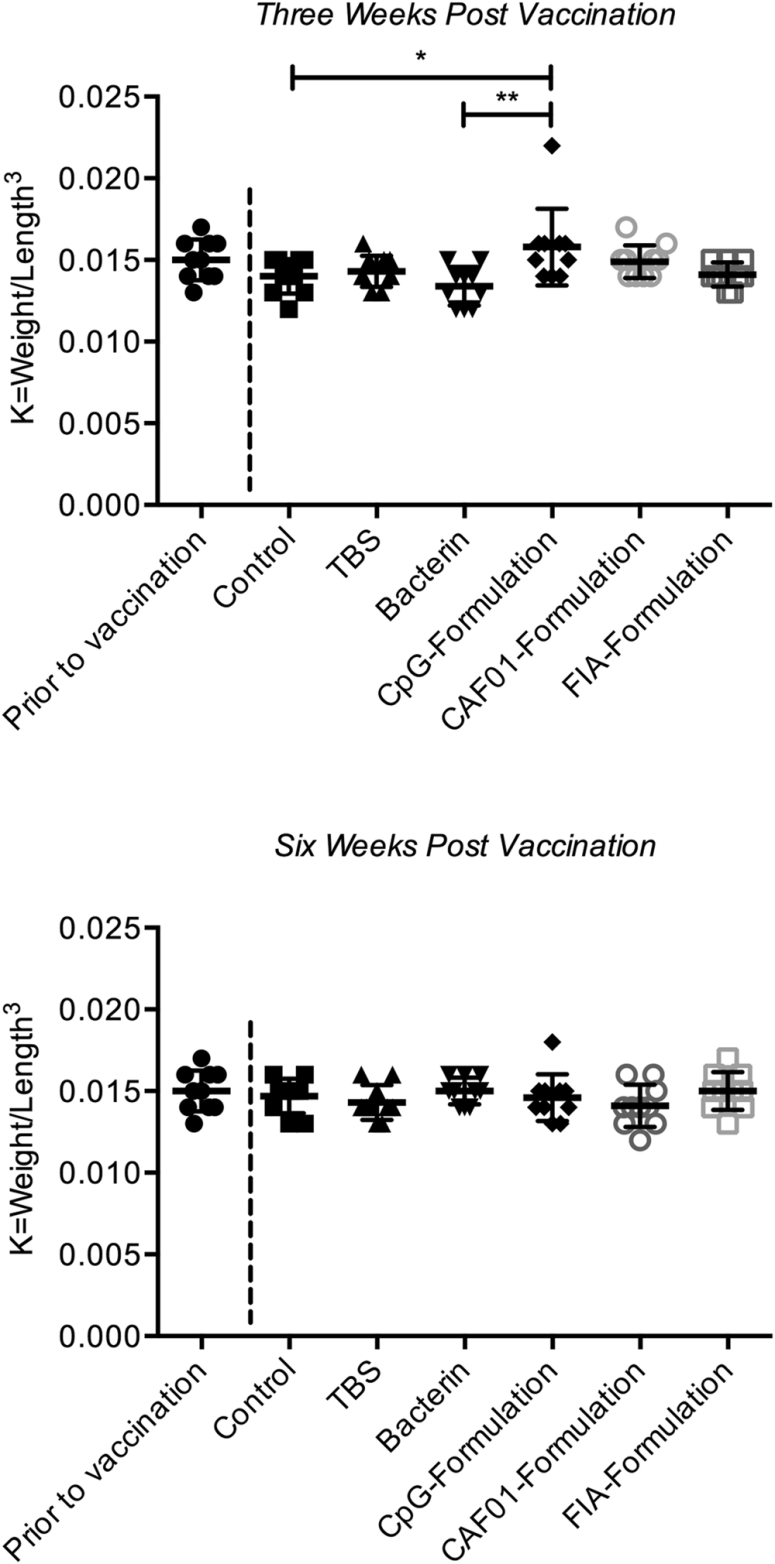
Fultons condition factor. Calculations and statistical methods are described in the materials and methods section. Values are presented as dot-plots with mean +/− standard deviation. Asterisks show statistically significant differences between groups and denote: *P < 0.05 and **P < 0.01. Condition factors obtained prior to vaccination are only for reference purposes. These were not included in the statistical comparisons.

To assess whether or not any of the vaccine formulations influenced the fitness or condition of the fish, Fultons condition factor was calculated and recorded for a sampled subset of each group at three different time points. Comparing the groups within each sampling time point, only the CpG-formulation displayed a statistically significant difference from the naïve control group, as well as the bacterin group at three weeks post vaccination (Fig. 1).

### Assessment of Subclinical Infections

The analyses of collected spleen samples were all negative for each of the seven pathogens addressed. Since samples were taken from each of the included experimental groups, and cover all included holding tanks, the fish used in this experiment were regarded as free of those pathogens.

### Gross pathological examination

**Figure 2:**
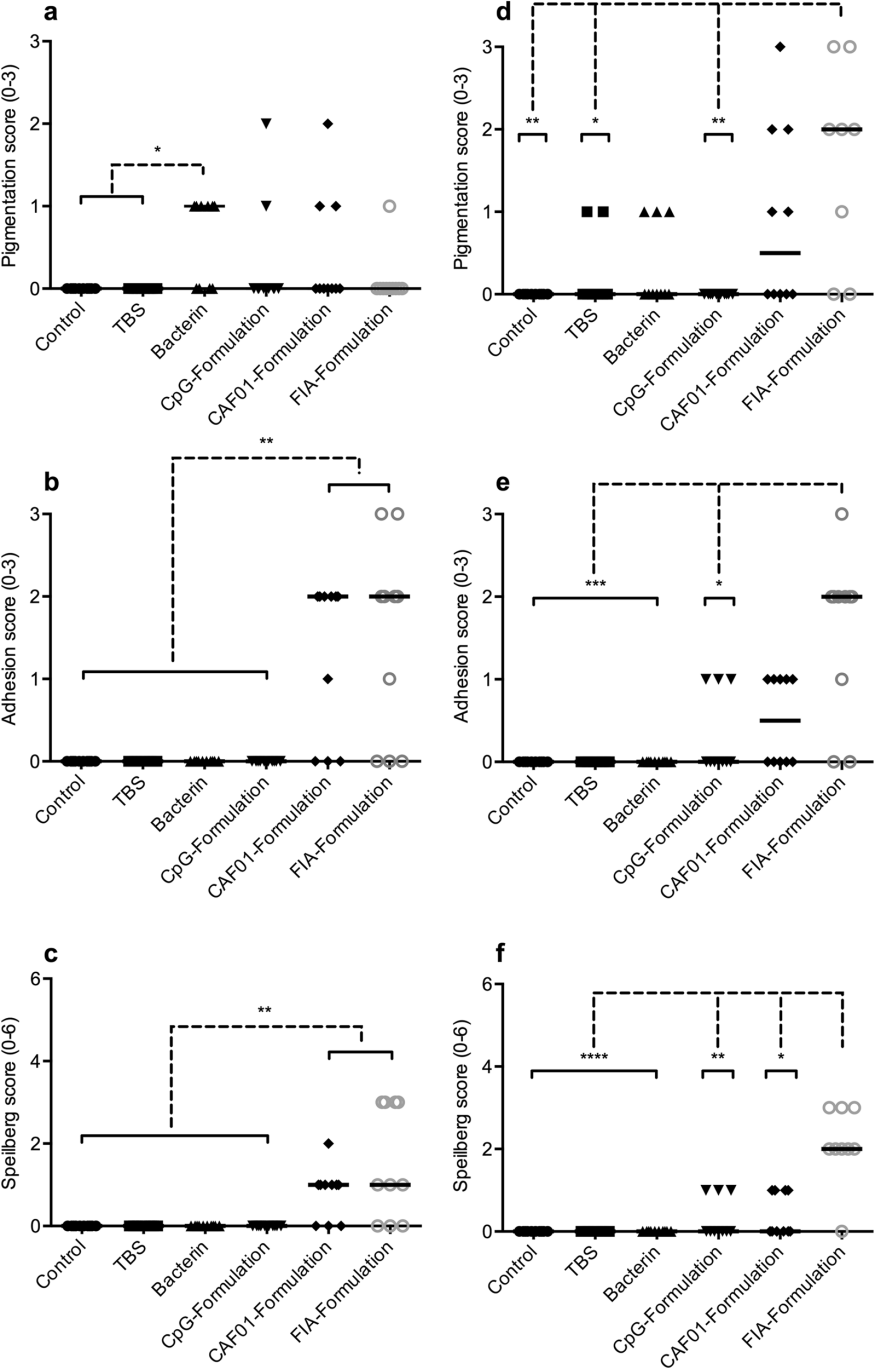
Results from post-mortem gross pathological scoring performed three (**a**–**c**) and ten (**d**–**f**) weeks post vaccination. Scoring and statistical methods are described in the materials and methods section. Scores are presented as dot-plots with median scores indicated. Asterisks show statistically significant differences between groups and denote: *P < 0.05, **P < 0.01, ***P < 0.001 and ****P < 0.0001.

**Figure 3:**
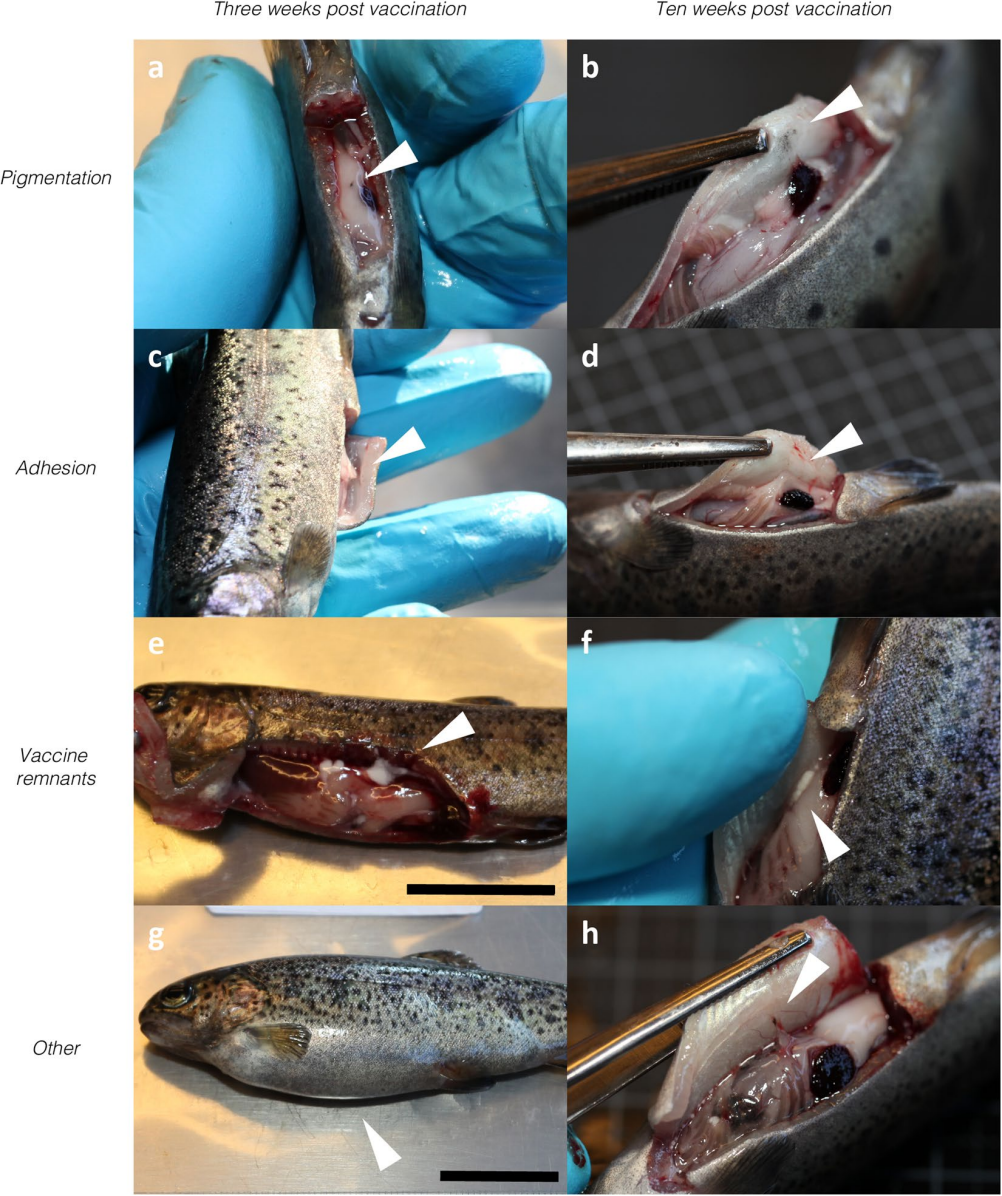
Representative macroscopic observations made during post-mortem examination of vaccinated individuals at three and ten weeks post vaccination, shown on the left (**a**,**c**,**e** and **g**) and right hand side (**b**,**d**,**f** and **h**), respectively. Arrowheads indicate each observation. A) Focal pigmentation of intraperitoneal adipose tissue, bacterin group. (**b**) Diffuse pigmentation of abdominal viscera, CAF01 group. (**c**) Adhesion connecting adipose tissue to injection site, CAF01 group. (**d**) Adhesion connecting adipose tissue to injection site, CPG group. (**e**) Remnants of FIA emulsion in peritoneal cavity, FIA group. (**f**) Remnants of FIA emulsion in peritoneal cavity, FIA group. (**g**) Abdominal distension, CpG group. (**h**) Pigmentation near injection site, adhesion connecting viscera to injection site, remnants of FIA emulsion scattered in peritoneal cavity, FIA group. Scale bars measure 2 centimeters.

The gross lesions registered following post-mortem examination are shown in Fig. 2. Representative observations are shown in Fig. 3. Occurrences within a group are quantified as the fraction of occurrences (O) in X examined individuals (O/X). No gross pathological changes were recorded at either time point in the control group.

#### TBS group

*Three weeks post vaccination:* No gross pathological changes were recorded. *Ten weeks post vaccination:* Single, focal pigmented changes were observed in intraperitoneal adipose tissue beneath the site of injection in 2/10 individuals.

#### Bacterin group

*Three weeks post vaccination:* Single, intraperitoneal, focal pigmented changes were observed in 6/10 individuals (see Fig. 3a). The pigmented changes were predominantly found in adipose tissue beneath the site of injection, and the pigmentation scores were statistically significantly higher than those of the control and TBS groups (P < 0.05). *Ten weeks post vaccination:* Single, focal pigmented changes were found in intraperitoneal adipose tissue beneath the site of injection in 3/10 individuals.

#### CpG group

*Three weeks post vaccination:* Abdominal distension due to an accumulation of cloudy, white fluid was observed in 9/10 individuals, (see Fig. 3g). Focal, pigmented changes in intraperitoneal adipose tissue was found in 2/10 individuals, with one scored as single pigmentation, and the other as moderate pigmentations due to differences in severity. *Ten weeks post vaccination:* No abdominal distension was observed at this time point. Single adhesions connecting injection site and intraperitoneal viscera were observed in 3/10 individuals (Fig. 3d).

#### CAF01 group

*Three weeks post vaccination:* Pigmented changes of intraperitoneal adipose tissue were observed in 3/10 individuals. In two of these, single, focal pigmented changes were observed and in the third, moderate pigmentation was found. In 1/10 individuals, a focal, dark coloration was observed in the liver. Adhesions connecting the site of injection with the abdominal viscera were observed in 7/10 individuals, with a single adhesion observed in one of these individuals, while the remaining six showed moderate adhesions. A representative example is shown in Fig. 3c. Adhesion scores were statistically significantly higher than those of all groups but the FIA group (P < 0.01). The recorded Speilberg scores were statistically significantly higher than those of all groups but the FIA group (P < 0.01) *Ten weeks post vaccination:* Pigmented changes of the abdominal viscera were observed in 5/10 individuals, ranging from single to extensive pigmentations, and found in adipose tissue as well as in musculature (see Fig. 3b). Single adhesions were observed in 5/10 individuals.

#### FIA group

*Three weeks post vaccination:* A single, focal pigmented change in intraperitoneal adipose tissue was observed in 1/10 individuals. Intraperitoneal adhesions were observed in 7/10 individuals. In one of these, a single, but well organized adhesion was observed, while four individuals were found to have moderate adhesions, with two described as strong. Finally, extensive adhesions were observed in two of these individuals. The adhesion scores were statistically significantly higher than those of the control, TBS, bacterin and CpG group (P < 0.01). The recorded Speilberg scores were statistically significantly higher than those of all groups but the CAF01 group. In 7/10 individuals, droplets of emulsified vaccine were observed within the peritoneal cavity, as seen in Fig. 3e. *Ten weeks post vaccination:* Intraperitoneal pigmented changes were observed in 6/10 individuals, with a single pigmented change observed in one of these individuals, while moderate and extensive pigmented changes were described from three and two of these individuals, respectively. The pigmentation scores were statistically significantly higher than those of the TBS group (P < 0.05), as well as those of the control and CpG groups (P < 0.01). Intraperitoneal adhesions were described in 8/10 individuals, with a single adhesion observed in one of these, moderate adhesions in six, and extensive adhesions in the final one. Several of these were observed as strong, and in three of them the adhesions were not confined to the site of injection. The adhesion scores at this time point were statistically significantly higher than those of the control, TBS and bacterin groups (P < 0.001), as well as those of the CpG group (P < 0.05). The Speilberg scores were significantly higher than those of all other groups (P < 0.05). Furthermore, vaccine emulsion was observed in the peritoneal cavity of 7/10 individuals, as seen in Fig. 3f and h.

### Histopathology

**Figure 4:**
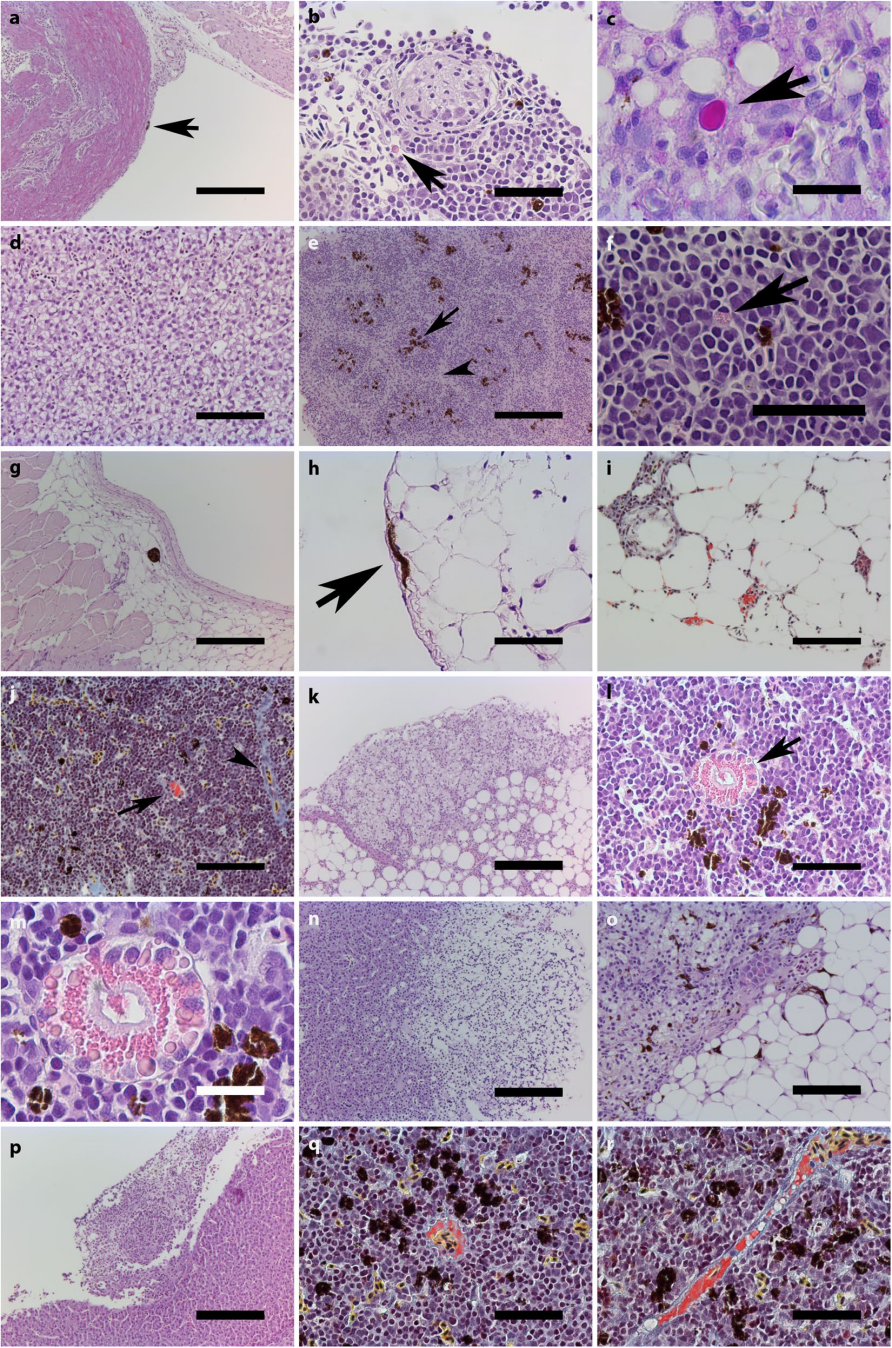
Histological examination of sampled tissues. Unless otherwise stated, samples are HE-stained. (**a**) Melanomacrophage(s) in bulbus arteriosus, FIA group, three weeks post vaccination (3 wpv). Scalebar (sb)=200 µm. (**b**) Mott cell in anterior kidney, control group, 3 wpv. Sb = 100 µm. (**c**) Mott cell in liver, PAS stained, 10 wpv. Sb = 20 µm. (**d**) Hepatocellular vacuolization, 3 wpv. Sb = 100 µm. (**e**) Clear distinction between red (arrowhead) and white (arrow) splenic pulps with melanomacrophages in white pulp, CpG group, 10 wpv. Sb = 200 µm). (**f**) Eosinophilic granule cell in anterior kidney, FIA group, 10 wpv. Sb = 50 µm. (**g**) Melanomacrophage(s) in extraperitoneal adipose tissue, TBS group, 10 wpv. Sb = 200 µm. (**h**) Melanomacrophages in intraperitoneal adipose tissue, bacterin group, 3 wpv. Sb = 50 µm. (**i**) Fibrin deposits in intraperitoneal adipose tissue, MSB stain, bacterin group, 3 wpv. Sb = 100 µm. (**j**) Fibrin deposits in anterior kidney. Fibrin stain (arrow), as well as collagen and erythrocytes (arrowhead) are seen, MSB stain, CpG group, 3 wpv. Sb = 100 µm. (**k**) Inflammation in intraperitoneal adipose tissue, CAF01 group, 3 wpv. Sb = 200 µm. (**l**) Apparent liposomes in anterior kidney, CAF01 group, 3 wpv. Sb = 50 µm. (**m**) Higher magnification of L. Sb = 20 µm. (**n**) Hepatic necrosis, CAF01 group, 10 wpv. Sb = 200 µm. (**o**) Inflammation in spleen, CAF01 group, 10 wpv. Sb = 100 µm. (**p**) Inflammation in liver, FIA group, 3 wpv. Sb = 100 µm. (**q**) Fibrin deposits in anterior kidney, MSB stain, FIA group, 3 wpv. Sb = 50 µm. (**r**) Fibrin deposits in anterior kidney, MSB stain, FIA group, 10 wpv. Sb = 50 µm.

Figure 4 shows both representative, as well as unique observations from the histological examination of the sampled tissues. In this material, four general observations were made: I) Except for in a few individuals, varying numbers of melanomacrophages were observed in the visceral pericardium of the *bulbus arteriosus* (Fig. 4a). II) Large cells with excentric, flattened nuclei, were present in all groups included in this study. Based on these characteristics, as well as their staining properties in HE and PAS stained sections, these appear to be Mott cells (Fig. 4b,c). III) The numbers of melanomacrophages and Mott cells appear to increase between three and ten weeks post vaccination. IV) Varying degrees of hepatocellular vacuolization was observed across all groups (Fig. 4d). No apparent pattern was observed with regard to specific groups, in part due to distinct variation within groups.

As with the macroscopic observations, the number of specific occurrences (O) in X examined individuals is given as the fraction O/X. The recorded observations for each group were as follows:

#### Control group

*Three weeks post vaccination*: Melanomacrophages were observed in the visceral surfaces of liver and abdomen of 2/10 individuals. Mott cells were generally observed in anterior kidneys and spleens. Spleen sections allowed a clear distinction between red and white pulp (see Fig. 4e for example). *Ten weeks post vaccination:* Melanomacrophages were observed in the abdominal viscera of 1/10 individuals, in gall bladder of 2/10 individuals, as well as in extraperitoneal adipose tissue of 2/10 individuals. Staining with MSB resulted in red stains within the vasculature of the anterior kidney of 2/5 individuals, indicating deposition of fibrin conformal with thrombosis. Mott cells were generally observed in anterior kidneys and spleens, as well as in the livers of 2/10 individuals. In 1/10 samples, an eosinophilic granule cell (EGC, see ref. 30) was observed in spleen (see Fig. 4f for example). Spleen sections generally revealed a less clear distinction between the red and white pulp.

#### TBS group

*Three weeks post vaccination:* In general, Mott cells were found in spleens, and to a lesser extent in anterior kidneys. In 2/10 individual, EGCs were observed in spleen. Distinction between red and white pulp was generally less distinct. *Ten weeks post vaccination:* Melanomacrophages were observed in intraperitoneal (2/10), as well as extraperitoneal adipose tissue (1/10, Fig. 4g), and gallbladder (1/10). Fibrin deposits were seen in the spleen of 1/5 individuals, as revealed by MSB staining. Mott cells were generally observed in anterior kidneys and spleens, and in 1/10 individuals an EGC was observed near a splenic ellipsoid. In general, a clear distinction was found between red and white pulp in spleens.

#### Bacterin group

*Three weeks post vaccination:* Melanomacrophages were observed in intraperitoneal (3/10) (Fig. 4h) and extraperitoneal adipose tissue (1/10). MSB staining revealed extensive fibrin deposition in intraperitoneal adipose tissue in 2/5 individuals, suggesting thrombosis (Fig. 4i). Mott cells were generally observed in anterior kidney, and in particular in spleen, where high numbers were observed in individual samples. Furthermore, in 1/10 individuals, an EGC was observed near a splenic ellipsoid. Generally a clear distinction between red and white pulp was found in spleens. *Ten weeks post vaccination:* Melanomacrophages were found immediately beneath the abdominal viscera in 3/10 individuals. Fibrin deposits indicating thrombi were observed in MSB stained sections from the liver of 1/5 individuals, and from the anterior kidney of an additional 2/5 individuals. Mott cells were observed in anterior kidneys, and in particular in spleens. In general, red and white pulps were easily distinguished, and melanomacrophages (4/10), Mott cells (3/10) and EGCs (2/10) were observed within the white pulp of spleens.

#### CpG group

*Three weeks post vaccination:* In 1/10 individuals, melanomacrophages were recorded in intraperitoneal adipose tissue, and in extraperitoneal adipose tissue in another 2/10, MSB staining revealed fibrin deposition indicating thrombosis in the anterior kidneys of 3/5 individuals (Fig. 4j). Mott cells were generally observed in anterior kidney and spleen. The distinction between red and white pulps varied throughout the group, but in several individuals the distinction was unclear. In 2/10 individuals, a number of melanomacrophages were observed immediately surrounding the ellipsoids. *Ten weeks post vaccination:* Melanomacrophages were observed in both intraperitoneal (1/10) and extraperitoneal (4/10) adipose tissues. Spleen structure showed some variation, however, red and white pulps were generally not easily differentiated. Furthermore, Mott (4/10) cells and melanomacrophages (3/10) were observed immediately surrounding the ellipsoids. EGCs were also observed in the spleen (4/10), and both Mott cells and EGCs were observed in anterior kidney, in general.

#### CAF01 group

*Three weeks post vaccination:* Melanomacrophages were observed in intraperitoneal (5/10), and extraperitoneal adipose tissue (1/10). In 1/10 individuals, a neutrophil was observed in intraperitoneal adipose tissue, and inflammation of the intraperitoneal adipose tissue was observed in 1/10 individuals, unrelated to the former individual (Fig. 4k). Furthermore, MSB staining showed fibrin deposition in intraperitoneal adipose tissue of 1/5 individuals. In general, Mott cells were observed in both spleens and anterior kidneys. In spleens, distinction between red and white pulps was difficult, and while melanomacrophages and Mott cells were observed within spleens, melanomacrophages were only recorded surrounding the ellipsoids in 1/10 individuals. In 1/10 individual, a circular arrangement of eosinophilic spheres in varying sizes was observed (Fig. 4l,m). *Ten weeks post vaccination:* Melanomacrophages were observed in abdominal viscera (3/10), intraperitoneal adipose tissue (3/10) and, in 1/10 individuals, in the liver. In two separate individuals, focal necrosis of the liver (1/10) (Fig. 4n) and an increased presence of white blood cells (white pulp enlargement compared with the size of red pulp) (1/10) was found (Fig. 4o), respectively. A decreased distinction between red and white pulps was observed across the samples. EGCs and Mott cells were generally observed in the anterior kidneys and spleens. In individual samples, EGCs (2/10), a Mott cell (1/10) and melanomacrophages (5/10) were found immediately next to ellipsoids. Unrelated observations of small, eosinophilic spheres were made in anterior kidney (1/10), as well as, in ellipsoids (1/10).

#### FIA group

*Three weeks post vaccination:* Melanomacrophages were observed in intraperitoneal adipose tissues (4/10), and in 1/10 individuals, inflamed tissue was observed on the visceral surface of the liver (Fig. 4p). MSB staining revealed fibrin deposition indicative of thrombosis in the anterior kidneys of 2/5 individuals (Fig. 4q). Mott cells were observed in anterior kidneys and in high numbers in the spleens in general. In 1/10 of these individuals, a Mott cell was observed within an ellipsoid. In 3/10 individuals, EGCs were observed in the spleen, as well. Distinctions between red and white pulps were clear, and melanomacrophages were observed immediately surrounding ellipsoids in 3/10 individuals. *Ten weeks post vaccination:* Fibrin deposits indicating thrombi were observed in MSB stained sections from the anterior kidneys of 2/5 individuals (Fig. 4r). Widespread presence of melanomacrophages was observed in adipose tissue directly associated with the pyloric ceca of 2/10 individuals. In addition to this, Mott cells (1/10) were observed in intraperitoneal adipose tissues, as well. In addition to that, Mott cells were generally observed in anterior kidney and spleen. A slightly decreased distinction between red and white pulps was found, and observations of EGCs (4/10) and melanomacrophages (4/10) in white pulp were made. The general presence both of these cell types was generally found to be elevated.

### Experimental infection study

**Figure 5:**
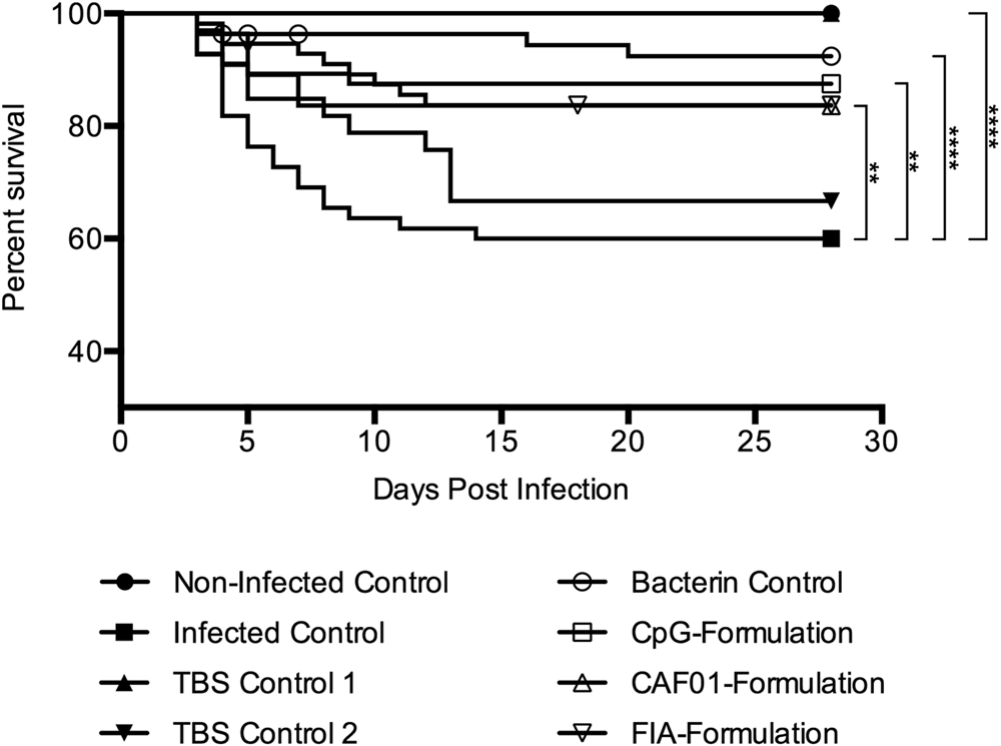
Results from experimental infection experiment. Methodology, calculations and statistical methods are described in material and methods section, as well as in the results section. Each graph is indicated by a symbol at 28 days post infection. Symbols prior to this indicate censored individuals (see materials and methods section). Asterisks show statistically significant differences between groups and denote: *P < 0.05, **P < 0.01, ***P < 0.001 and ****P < 0.0001.

The efficacy of each experimental vaccine was assessed by homologous, waterborne experimental infection. When tested, the duplicates of all groups, except for those of the TBS group (P = 0.0017), supported the null-hypothesis, that there was no difference between the tested duplicates, and thus the results from all groups, except for the TBS group, were combined into a single data set per group prior to further analysis. The collected results from the infection are shown in Fig. 5, and individual curves with 95% confidence intervals are shown in Supplementary Fig. S2. Mortalities began to occur 3 days post infection in all groups where mortalities were observed. For the infected controls, mortalities continued until 14 days post infection. For the CAF01, CpG and FIA groups, mortalities stopped at 7, 9 and 12 days post infection, respectively. As opposed to the more steady decrease in survival observed for the aforementioned groups, mortalities in the bacterin control group were scattered between day 3 and 20.

When comparing the survival curves for the non-immunized controls, there were statistically significant differences between those of the non-infected control group and the infected control group (P < 0.0001), and those of the TBS 2 replicate and the non-infected control group (P < 0.0001). As for the protective effects of immunization, the CpG (P = 0.0015), CAF01 (P = 0.006) and FIA group (P = 0.0037), as well as the bacterin control group (P < 0.0001) were all statistically significantly different from the infected control group, demonstrating a significantly reduced mortality in these groups. There were no statistically significant differences among those four groups. Table 3 shows the RPS and hazard ratio of each group when compared to the infected control group. The hazard ratios of the bacterin control, CpG, CAF01 and FIA group are all below 0.4, with their upper limit of their 95% confidence intervals all below 0.75, indicating a clear reduction in relative risk of mortality, when compared to that of the infected control group.

**Table 3:** Relative percent survival and hazard ratio results from the experimental infection.

Group:	Hazard Ratio:			
	RPS:	Ratio:	95% CI (Lower Limit)	95% CI (Upper Limit)
Bacterin Control	81.1%	0.16	0.10	0.44
CpG	68.8%	0.28	0.14	0.62
CAF01	59.1%	0.36	0.18	0.74
FIA	59.3%	0.34	0.17	0.70

## Discussion

In order to examine potential alternatives to mineral oil-adjuvanted vaccine formulations according to the criteria stated in the introduction, the *in vivo* performance of three experimental furunculosis vaccine formulations was assessed in rainbow trout. Given the production context, as well as the ethical considerations for exploring vaccines with improved safety profiles, the condition of the fish was addressed post vaccination. In previous studies, vaccine formulations utilizing aluminum salt and mineral oil adjuvants have been connected with growth reduction in Atlantic salmon^7, 30, 31^. Such reductions, however, were not seen in the present study, using Fulton’s condition factor as a measure. The only difference seen, although statistically significant, is most likely attributed to the fact that abdominal distension, caused by accumulated intraperitoneal fluid, was found in nine out of ten examined individuals in the CpG group. Furthermore, a reduced weight and/or reduced fitness would result in a decreased condition factor, whereas the CpG group shows an increase in condition factor three weeks post vaccination. Study length also plays a role, since the reduction seen in the previous studies were long-term reductions, while the condition factor in the present study was determined at much earlier time points.

Micro- and macroscopic examinations of individuals from each experimental group revealed changes in vaccinated individuals, as well as in some control group individuals observed as varying degrees of pigmented changes and adhesions observed during post-mortem examination. Pigmentation is due to the presence of melanomacrophages, occurring whenever such cells are present, however, their nature and modes of action are largely unknown^32, 33^, although they seem to be prominent in granulomatous inflammatory conditions^34^. Focal, melanized pigmentation of intraperitoneal adipose tissue directly beneath the site of injection was found in all injected groups, while adhesions were only observed in groups injected with bacterin formulated with adjuvants, and the dynamics, with respect to severity and occurrence, in each group varied. Where observations of pigmentation seemed to decrease over time for the bacterin- and CpG group, the opposite was observed for the CAF01 group, and for the FIA group in particular. For observations of adhesions, a decrease over time was seen for the CAF01 group, while occurrence and severity for the FIA group appeared steady. Besides differences in respective reactogenicity, these differences could likely be rooted in the physical properties of the injected material. While the observations of pigmentation in TBS injected fish indicate sensitivity to the procedure alone (potential inflammatory response to trauma), a key difference was that the bacterin and the CpG formulation were not particulate, as was the case with both the CAF01 (liposomes) and FIA (emulsions) formulations. Thus, the initial free load of inactivated bacteria will be higher immediately following administration, allowing a rapid processing of the total, injected load^35^. This might also, at least in part, explain the early abdominal distension with the CpG adjuvanted vaccine, leading to an increased systemic inflammation early after immunization, however, further studies would be required to verify this.

Particulate vaccine formulations offering a depot effect would offer a prolonged presence and a different release dynamic, changing the timeframe of the pathological changes, which could explain the differences seen in this study. The repeated observations of vaccine remnants ten weeks post vaccination in the FIA group, emphasize the prolonged effect of such emulsions, which is assumed to contribute to the overall reactogenicity of FIA previously shown for FIA injected without antigenic content in rainbow trout^5^, as well as for similar mineral-oil based vaccine formulations in other salmonid fishes^36, 37^.

When comparing the observations of pigmentation and adhesion to the scoring criteria of the Speilberg score^3^, a disagreement concerning the relative weight of pigmentation is seen. While focusing on adhesions, melanized pigmentation is only described in the criteria for the two most severe scores (scores 5–6) of the Speilberg score. This does not correspond well with the observations made in this study, as pigmentation occurs independently of adhesions, most likely reflecting a difference in adverse reactions between species^37^, since the Speilberg score was initially used for scoring adverse reactions in salmon.

The CpG formulation consistently scored low in all three scoring systems, however, the abdominal distension observed three weeks post vaccination was not covered by any of the three. Besides the effect of a potential systemic infection discussed earlier, other causes for accumulation of fluid in the abdomen include peritonitis and ascites caused by pathological changes in the liver, yet histopathological examinations of individuals from the CpG group did not reveal any changes in this organ. MSB staining did, however, indicate thrombosis in anterior kidney capillaries in the CpG group, and it is considered plausible that any form of vessel occlusions could interfere with the homeostasis in the circulatory system, possibly leading to oedema and ascites. The early findings of fibrin deposition in all groups injected with bacterin, either alone or formulated with an adjuvant, would suggest that the use of whole-cell bacterin could result in thrombosis. Similar deposits were previously found in rainbow trout, as well as in Atlantic salmon intraperitoneally injected with mineral oil-adjuvanted vaccines^5, 9^. While the findings of fibrin in control and TBS group individuals at ten weeks post vaccination indicated that a certain background level of fibrin deposits could be expected, fibrin deposits appeared at an earlier time point, located in adipose tissue close to the injection site. This suggests that the bacterin and vaccines, if not initiates, then at least accelerates the changes seen for those groups, although further studies would be needed to investigate and verify this. Besides suggesting endothelial damage of the vascular system, occlusive thrombosis must be considered a significant disadvantage due to potential tissue damage as a consequence of restricted blood flow. In all injected groups, melanomacrophages were observed in intraperitoneal adipose tissue and/or immediately beneath the abdominal viscera near the injection site. This appears to support the post-mortem findings regarding macroscopic pigmentation, and furthermore, it substantiates the idea of a general sensitivity towards the injection itself.

Regarding the dynamics of particular cell types, the observations of Mott cells across all groups are surprising. Mott cells in fish were described by Haugarvoll *et al*.^38^ as dysregulated plasma cells, and have previously been associated with vaccine-induced adverse effects in Atlantic salmon. While there appears to be a general increase over time, and while the FIA group in particular was found to contain high numbers of this cell type, the uniform pattern of observations in spleen and anterior kidney was consistent throughout all groups. The fact that melanomacrophages were observed immediately adjacent to ellipsoids in the splenic white pulp in each vaccine group at three weeks post vaccination might reflect an induced state of alertness that is supported by the appearance of Mott cells and EGCs at ten weeks post vaccination, but this remains speculative at this point. Furthermore, the supposed presence of CAF01 liposomes in one of the major teleost lymphoid tissues implies that the vaccine is taken up and processed efficiently in a lymphocyte-rich environment. The observations of inflammation or even necrosis of the viscera of spleen, adipose tissue and liver in groups receiving emulsified formulations, however, support the previous findings of sensitivity towards long-term exposure to intraperitoneally administered vaccines in salmonids^36, 37^.

In terms of the vaccine-induced adverse effects observed in the present study, those observed in the FIA group were expected based on the previous study using the same formulation, as well as a similar, commercially available formulation^5^, justifying its use as the benchmark formulation. Previous experiences with CpG oligodeoxynucleotides in various species of fish have been summarized by Carrington & Secombes^39^. In their respective studies, Rhodes *et al*. did not observe any pathological changes after intraperitoneal injection of CpG oligodeoxynucleotide-based formulations in Chinook salmon (*Oncorhynchus tshawytscha*)^40^, and Carrington *et al*. mention that no adverse effects were found after intramuscular administration of CpG-adjuvanted *A. salmonicida* bacterin in rainbow trout^23^. These observations contrast those of the present study, where pigmentation, adhesions and abdominal distension, in particular, was observed. Since this is the first study on the use of CAF01 in fish, the expectations were based on the safety profile reported from use in mammals^18^. Overall, while the use of whole-cell bacterin likely imparts a certain level of adverse reactions on its own, neither of the three experimental formulations offer a complete elimination of adverse effects. While the FIA-formulation in particular results in a certain level of reactogenicity, some reductions in histopathological changes, as well as statistically significant reductions in gross pathological scores, were seen for both the CpG- and the CAF01-formulations.

Besides the adverse reactions to the experimental vaccines, the other main objective of this study was to assess the induced protection from the experimental infection. The absence of mortality in the TBS 1 aquarium is attributed to an allocation error resulting in a lower number of individuals, leading to a decrease stress from crowding, and a presumed decrease in subsequent horizontal spread of infection. Since an increased number of individuals were consequently allocated to the TBS 2 aquarium, the results from these two tanks should be interpreted with caution.

All vaccine formulations, as well as the bacterin alone, were shown to successfully confer protection against waterborne infection, as seen from the Kaplan-Meier survival curves, as well as from the substantial risk reduction seen in the calculated hazard ratios. Despite the apparent success of the bacterin injections, the protection is believed to be due to the relatively short experimental timeframe, even though the experimental infection was performed more than 1200 degree days post vaccination, well beyond the 400–600 degree days typically required for successful induction of protection in similar, commercial vaccines. In the context of a full production cycle (>9 months according to FAO^41^), satisfactory protection will require long-term protection beyond the timeframe of this study. The protection induced by the unadjuvanted bacterin injection is somewhat surprising, given that Midtlyng *et al*. saw no protection from unadjvanted *A. salmonicida* vaccines in Atlantic salmon during co-habitation infection experiments at six weeks, three months and six months post vaccination^3^, making it harder to assess the added protection induced by each adjuvant. Benefits from using adjuvants like CpG and CAF01, both when it comes to pathological score and efficacy, will most likely be more visible in combination with purified/recombinant subunit antigens instead of whole cells, most likely also containing immunostimulatory molecules.

The results from the present study show that vaccines formulated with either CpG ODN’s or the liposomal CAF01 adjuvant offer reductions in adverse effects relative to the benchmark FIA formulation, and seemingly comparable levels of protection. The two particulate formulations CAF01 and FIA generally recorded the highest macroscopic pathological scores, while the bacterin and CpG groups recorded lower scores. This picture, however, grows more complicated when the abdominal distension and histopathological observations are taken into consideration. While neither the CpG ODN nor the CAF01 adjuvant completely eliminates adverse effects, they are, however, considered less harmful relative to mineral-, or paraffin oil adjuvants^5^, based on the observations presented in this study. It should be noted that the experimental vaccine formulations included in this study includes antigens from only one pathogen. Multivalent vaccines such as those used in commercial aquaculture are more complicated and synergies between multiple pathogens have previously been reported^42^. The basic formulation, however, is still considered comparable to that of the benchmark formulation included in this study.

While adjuvants can provide a major contribution to the overall reactogenicity of vaccine formulations, the apparent sensitivity towards the injection alone and adverse effects such as thrombosis resulting from injection of whole-cell bacterin seen in this study, will likely make an elimination of adverse effects very difficult to accomplish. Further studies into vaccine formulations are needed and while identification and incorporation of recombinant protective antigens would make the use of a whole-cell bacterin obsolete, mineral oil adjuvants alone produce a number of adverse effects in rainbow trout^5^. Finding the correct balance between stimulation and overstimulation of the immune response will still be a vital key to future vaccines providing better protection, allowing optimized production and improving animal welfare.

## Electronic supplementary material

Supplementary information
