# Supplementary material for: Alternatives to mineral oil adjuvants in vaccines against Aeromonas salmonicida subsp. salmonicida in rainbow trout offer reductions in adverse effects
Source: Sci Rep. 2017 Jul 19;7:5930. doi: 10.1038/s41598-017-06324-7 (PMC5517504; doi:10.1038/s41598-017-06324-7)
Supplement: Supplementary file 1 — Supplementary information [file 41598_2017_6324_MOESM1_ESM.pdf]

Supplemental material for:

Alternatives to Mineral Oil Adjuvants in Vaccines against *Aeromonas salmonicida* subsp. *salmonicida* in Rainbow Trout Offer Reductions in Adverse Effects

Kasper Rømer Villumsen[1$], Erling Olaf Koppang[2], Dennis Christensen[3] & Anders Miki Bojesen[1]

[1]*Department of Veterinary and Animal Sciences, University of Copenhagen, Denmark*

[2]*Faculty of Veterinary Medicine, Norwegian University of Life Sciences, Norway*

[3]*Adjuvant Research, Statens Serum Institut, Denmark*

[$] *Corresponding author: krv@sund.ku.dk*

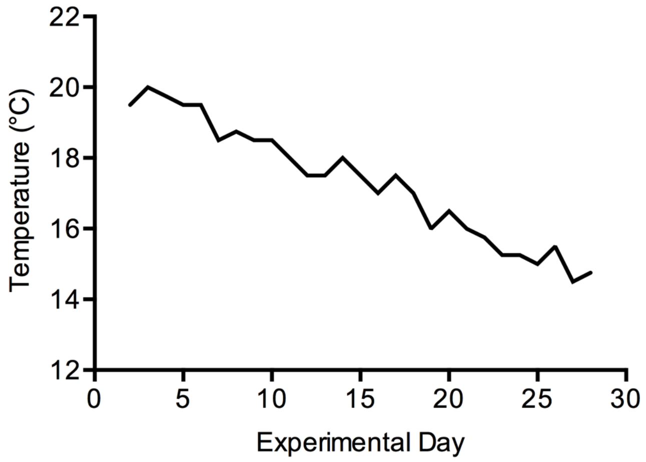

Supplementary Figure 1: Temperature curve for the experimental infection experiment. Temperatures are average water temperatures from separate tanks.

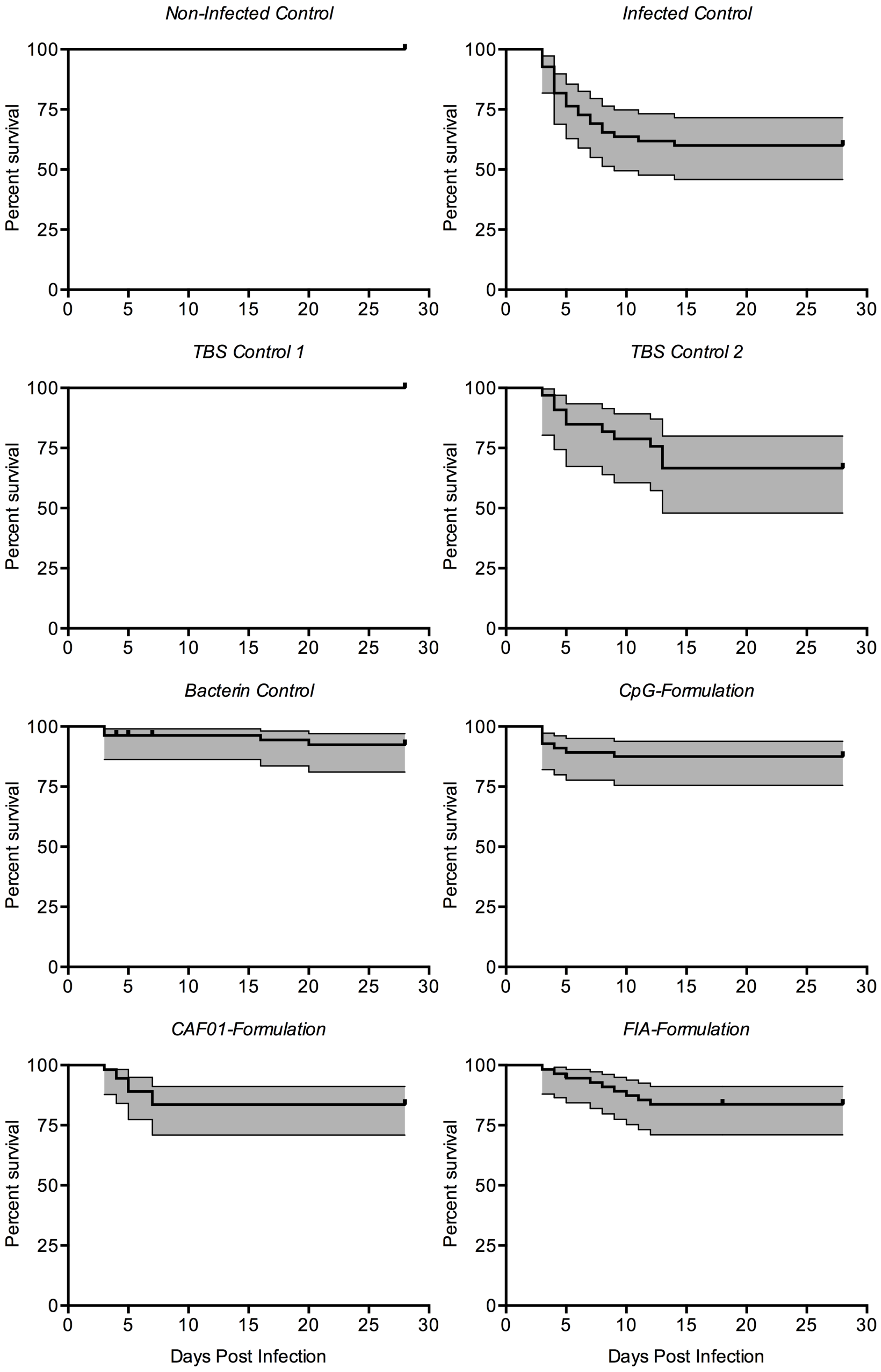

Supplementary Figure S2: Results from experimental infection experiment – Isolated. Methodology and calculations are described in material and methods section, as well as in the results section. A tick

at 28 days post infection concludes each graph. Ticks prior to this indicate censored individuals (see materials and methods section). Each graph is shown with its respective 95% confidence interval (gray shading).
